# Diffusion magnetic resonance imaging assessment of regional white matter maturation in preterm neonates

**DOI:** 10.1007/s00234-020-02584-9

**Published:** 2020-10-29

**Authors:** J. A. Kimpton, D. Batalle, M. L. Barnett, E. J. Hughes, A. T. M. Chew, S. Falconer, J. D. Tournier, D. Alexander, H. Zhang, A. D. Edwards, S. J. Counsell

**Affiliations:** 1 Centre for the Developing Brain, School of Imaging Sciences & Biomedical Engineering, King’s College London, London, UK; 2 Department of Forensic and Neurodevelopmental Science, Institute of Psychiatry, Psychology and Neuroscience, King’s College London, London, UK; 3 Department of Computer Science and Centre for Medical Imaging Computing, University College London, London, UK

**Keywords:** Neonate, Preterm, Tractography, Diffusion magnetic resonance imaging (dMRI), Neurite orientation dispersion and density imaging (NODDI), High-angular resolution diffusion MR imaging (HARDI)

## Abstract

**Purpose:**

Diffusion magnetic resonance imaging (dMRI) studies report altered white matter (WM) development in preterm infants. Neurite orientation dispersion and density imaging (NODDI) metrics provide more realistic estimations of neurite architecture in vivo compared with standard diffusion tensor imaging (DTI) metrics. This study investigated microstructural maturation of WM in preterm neonates scanned between 25 and 45 weeks postmenstrual age (PMA) with normal neurodevelopmental outcomes at 2 years using DTI and NODDI metrics.

**Methods:**

Thirty-one neonates (*n* = 17 male) with median (range) gestational age (GA) 32^+1^ weeks (24^+2^–36^+4^) underwent 3 T brain MRI at median (range) post menstrual age (PMA) 35^+2^ weeks (25^+3^–43^+1^). WM tracts (cingulum, fornix, corticospinal tract (CST), inferior longitudinal fasciculus (ILF), optic radiations) were delineated using constrained spherical deconvolution and probabilistic tractography in MRtrix3. DTI and NODDI metrics were extracted for the whole tract and cross-sections along each tract to assess regional development.

**Results:**

PMA at scan positively correlated with fractional anisotropy (FA) in the CST, fornix and optic radiations and neurite density index (NDI) in the cingulum, CST and fornix and negatively correlated with mean diffusivity (MD) in all tracts. A multilinear regression model demonstrated PMA at scan influenced all diffusion measures, GA and GAxPMA at scan influenced FA, MD and NDI and gender affected NDI. Cross-sectional analyses revealed asynchronous WM maturation within and between WM tracts.).

**Conclusion:**

We describe normal WM maturation in preterm neonates with normal neurodevelopmental outcomes. NODDI can enhance our understanding of WM maturation compared with standard DTI metrics alone.

**Supplementary Information:**

The online version of this article (10.1007/s00234-020-02584-9) contains supplementary material, which is available to authorized users.

## Introduction

The incidence of preterm birth is increasing, impacting around 12–13% of all live births in the United States of America (USA) and 5–9% in other developed countries [1]. Survivors of preterm birth have a high prevalence of neurodevelopmental impairments, including cognitive deficits, developmental coordination disorder and behavioural problems [2, 3]. Diffusion magnetic resonance imaging (dMRI) studies have identified altered white matter (WM) development in preterm infants [4, 5], which is associated with neurodevelopmental impairments [6, 7]. Increasing our understanding of WM microstructural development between birth and term equivalent age in preterm infants with normal neurodevelopment in early childhood may be informative to identify early markers of abnormal WM development and those at risk of adverse neurodevelopmental outcomes.

WM development begins in the foetal brain around 13–18 weeks post-conception with limbic, projection and commissural pathways, followed by thalamocortical and association pathways around 24–32 weeks and continues to rapidly develop during the first 2 years of life [8]. Histological studies have identified several distinct stages of WM development: axonal formation, neuronal and synaptic overproduction (preserving neuronal plasticity), elimination and pruning (creating more organised and streamlined networks), pre-myelination (the maturation of oligodendrocytes forming the myelin sheath) and finally myelination (the ensheathment of oligodendroglial processes around the axons to provide rapid and efficient conduction of nervous impulses improving brain connectivity) [9–11].

Whilst histological studies describing WM maturation are informative, they can only be performed ex vivo. dMRI and tractography enable quantitative measures of WM tract microstructure to be determined through non-invasive, in vivo imaging methods. These measures include fractional anisotropy (FA) (a measure of how restricted water molecular motion is in one direction compared with other directions) and mean diffusivity (MD) (the directionally averaged magnitude of water diffusion). Previous neonatal dMRI tractography studies have reported a negative correlation between FA and MD [12], increasing FA and decreasing MD with increasing age at scan [12–16] and preterm infants scanned at term have a higher MD compared with healthy term controls [17], as well as higher MD and lower FA in later childhood [18]. WM FA and MD may help to predict neurodevelopmental outcomes in preterm infants in early childhood [18–21] and neuropsychiatric conditions in later childhood, for example, autism spectrum disorder [22] and attention-deficit hyperactivity disorder [23].

To date, most studies assessing preterm brain development have used diffusion tensor imaging (DTI). However, there are two major drawbacks to DTI. First, DTI cannot resolve more than one fibre population in a voxel. To overcome this, high-angular resolution diffusion MR imaging (HARDI) tractography can be used in combination with constrained spherical deconvolution (CSD) to identify complex fibre configurations and orientations within each voxel and delineate WM tracts that traverse regions of crossing fibres [24]. Second, whilst DTI metrics are sensitive to WM changes, they lack specificity. For example, changes in FA may reflect many changes in several axonal properties including its diameter, dispersion, density, membrane permeability or myelination or even properties of non-white matter tissue types such as cerebrospinal fluid (CSF) [25]. Neurite orientation dispersion and density imaging (NODDI) is a more advanced imaging analysis technique that provides a three-compartment model that accounts for intra- and extracellular volume fractions, as well as CSF [26]. NODDI can discriminate between different components of WM microstructure and provide more specific markers of neurite architecture that are more biologically relevant than DTI metrics [26, 27] and consistent with histology [28]. For example, neurite density index (NDI) reflects myelination, axonal growth or axonal density, whilst orientation dispersion index (ODI) describes the dispersion of neurites, providing information about coherence and geometry. Both NDI and ODI contribute to the measured FA value in varying quantities. Increased FA is typically associated with increased NDI and decreased ODI [26]. NODDI metrics have been used to characterise WM microstructure in neonates [29, 30] and may be associated with neurodevelopmental outcomes [31] and intelligence quotient (IQ) [18] in childhood.

NODDI and DTI metrics can be used to assess WM maturation along an entire WM tract; however, this may mask important spatial changes in WM maturation within the tract. Regional analyses can be performed at regular intervals along WM tracts in cross-sectional planes to examine the homogeneity of WM development within the tract [32]. This concept has been used to identify age-related changes in healthy volunteers using DTI [33–37] and NODDI [38] but has not been performed in preterm infants scanned in the neonatal period.

The aim of this study was to investigate normal WM microstructural development between birth and term-equivalent age in a population of preterm infants with normal neurodevelopment in early childhood. We chose a range of WM tracts to represent limbic (cingulum and fornix), projection (corticospinal tract (CST), optic radiations) and association (inferior longitudinal fasciculus (ILF)) tracts. We obtained DTI and NODDI metrics for each WM tract, as well as at regular cross sections within each tract to assess regional maturation. We investigated how DTI and NODDI metrics changed with postmenstrual age (PMA) at scan, the relationship between DTI and NODDI metrics, factors that influenced diffusion measures within the tract (gestational age, PMA at scan, gender, type of tract, side of the tract) and regional differences in WM maturation.

## Methods

### Participants

This was part of a wider study investigating brain development in preterm infants and was conducted following Research Ethics Committee approval (09/H0707/98 and 12/LO/1247). Participants were prospectively recruited from the neonatal unit, and written parental consent obtained. Participants were included in the study if they were born < 37 weeks GA and excluded if they were > 46 weeks PMA at scan; had congenital malformations; there was severe motion artefact or focal lesions on MRI. The study cohort included MR images from 31 participants with a median (range) GA at birth of 32^+1^ weeks (range 24^+2^–36^+4^) and median (range) PMA at scan of 35^+2^ weeks (25^+3^–43^+1^) (Table 1).

**Table 1:** Clinical characteristics of the study population

Clinical characteristic	
Sex (male/female)	17/14
GA at birth (median, range) (weeks + days)	32^+1^ (24^+2^–36^+4^)
PMA at scan (median, range) (weeks + days)	35^+2^ (25^+3^–43^+1^)
Age at scan (median, interquartile range) (days)	10 (7–35)
More than 2 days of respiratory support (number, percentage)	9 (29%)
Necrotising enterocolitis (number, percentage)	3 (10%)
Birth weight (median, range)	1570 g (710–2940 g)
Birth weight < 10th centile (*n*)	7
Twins (*n*)	14
Bayley’s III scale for cognition (median, interquartile range)	95 (90–100)
Bayley’s III scale for motor (median, interquartile range)	97 (94–100)

### Magnetic resonance imaging acquisition

Neuroimaging data were acquired using a 3-Tesla Philips Achieva system (Best, The Netherlands) situated in the neonatal intensive care unit using a 32-channel head coil. MRI sequences included T1-weighted 3D MPRAGE (repetition time (TR) = 17 ms, echo time (TE) = 4.6 ms, flip angle 13°, voxel size 0.82 × 0.82 × 0.8 mm); T2 weighted fast spin echo (TR = 8670 ms, TE = 160 ms, flip angle 90°, slice thickness 2 mm with 1 mm overlapping slices, in plane resolution 1.14 × 1.14 mm) and diffusion MRI (2-mm isotropic resolution and SENSE factor of 2 in 2 shells; 64 non-collinear directions, *b* = 2500 s/mm^2^, 4 non-diffusion-weighted image (*b* = 0) with TR 9000 ms and TE 62 ms; 32 non-collinear directions, *b* = 750 s/mm^2^, 1 non-diffusion-weighted image (*b* = 0) with TR 9000 ms and TE 49 ms).

A paediatrician experienced in MR imaging attended all scans. Neonates < 37 weeks PMA at scan were scanned during natural sleep and > 37 weeks PMA at scan were sedated prior to scanning with oral chloral hydrate (25–50 mg/kg). Neonates had continuous monitoring during the scan including pulse oximetry, temperature and heart rate monitoring. Auditory protection was provided using earplugs moulded from a silicone-based putty (President Putty, Coltene, Whaldent, Mahwah, NJ, USA) placed in the external auditory meatus and neonatal earmuffs (MiniMuffs, Natus Medical Inc., San Carlos, CA, USA).

### Pre-processing

T2-weighted images were bias corrected [39], brain extracted and segmented into WM, grey matter, deep grey matter and CSF using a neonatal-specific algorithm [40]. The brain was parcellated using a block matching non-linear regression using a version of the standard anatomical automatic labelling (AAL) atlas adapted to the neonatal brain [29]. Parcellation of the atlas was propagated into each subject’s native space based on nearest neighbour propagation. In addition, an interhemispheric mask was delineated manually for each subject.

dMRI images were visually inspected for motion artefact, and corrupted volumes removed. Subjects included in the study had a maximum of 8 volumes (median 3, range 0–8) excluded from the higher shell, and 6 volumes (median 2, range 0–6) excluded from the lower shell. Volumes were corrected for EPI phase encoding distortions and eddy current–induced artefacts using FSL5.0 top up-eddy algorithm using a T2 volume rigidly registered to the b0 images and assuming a bandwidth of 0 (no phase encoding). This process was performed separately for the two acquired shells and their corresponding *b* = 0 volumes, and then the lower shell was rigidly registered to the averaged *b* = 0 volumes acquired with the higher shell. Gradient directions were rotated accordingly. All rigid registrations were performed with IRTK software.

### Tractography

To reconstruct the WM tracts, the higher shell data was used to estimate fibre orientation distribution (FOD) at each voxel using constrained spherical deconvolution (CSD) implemented in MRTrix3 (www.mrtrix.org) [24]. Whole-brain tractography was performed on the FODs using an anatomically constrained tractography (ACT) probabilistic algorithm [29], producing 100 million streamlines per subject. Specific WM tracts (cingulum, CST, fornix, ILF, and optic radiations) were segmented from the 100 million streamlines obtained for each participant using anatomically defined regions of interest from the parcellation previously calculated (Online Resource 1).

### Estimation of microstructural features

**Fig. 1:**
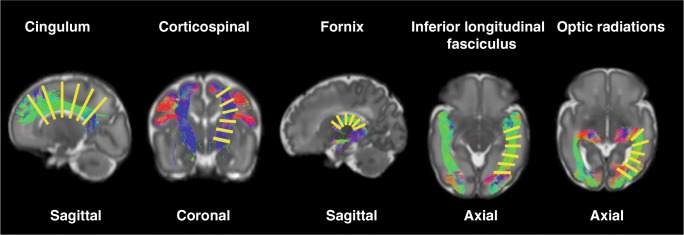
Location of the regional analysis in each of the segmented white matter tracts

DTI metrics (FA and MD) were obtained using MRTrix3 from the lower shell (*b* = 750 s/mm^2^). NODDI metrics (NDI and ODI) were obtained using the NODDI toolbox from a combination of the higher (*b* = 2500 s/mm^2^) and lower (*b* = 750 s/mm^2^) shells. Data were normalised by *b* = 0 volumes to account for differing TE/TR times between lower and higher shell [29]. DTI and NODDI metrics were obtained for each of the delineated WM tracts and at 6 equally spaced cross-sectional regions along each tract (Fig. 1) [32].

### Outcome data

Standardised neurodevelopmental assessment was carried out by an experienced paediatrician or developmental psychologist with the Bayley Scales of Infant and Toddler Development, Third Edition (BSID-III), at a median (range) corrected age of 25.5 months (24–32.5 months). Participants were included in the study if they achieved a score of 85 or more on both cognitive and motor categories.

### Statistical analyses

#### Correlation analysis

The relationship between PMA at scan with FA, MD, NDI and ODI in each tract was analysing using Pearson’s correlation in SPSS (v.24). Results were corrected for multiple comparisons using the Bonferroni correction for multiple significance testing; *p* < 0.00125 was considered significant, given that there were 5 tracts × 2 sides × 4 diffusion measures (0.05/40).

#### Multiple regression model

To analyse the contribution of different covariates (extremely/very preterm (GA < 32/40) vs moderate/late preterm (> 32/40 but < 37/40 GA), PMA at scan, gender, side of the tract, type of tract) on diffusion measures (FA, MD, NDI, ODI), a multiple regression with bootstrap was performed in Stata v.13. Results with *p* < 0.05 were considered significant.

Graphs were generated using R (www.r-project.org/) and PowerPoint. Tractography images were created using MRTrix3 (www.mrtrix.org) and PowerPoint.

## Results

### Association between PMA at scan and DTI metrics in each tract

Increasing PMA at scan was associated with decreasing MD and increasing FA and NDI. The relationship with ODI was less clear (Online Resources 2a-d, Table 2).

**Table 2:** Correlation analysis for PMA at scan and average diffusion measures for each tract

Side	Diffusion measure	Correlation coefficient				
		Cingulum	CST	Fornix	Inferior longitudinal fasciculus	Optic radiations
Left	FA	0.515	0.764*	0.783*	0.347	0.569*
	MD	− 0.625*	− 0.785*	− 0.789*	− 0.555*	− 0.735*
	NDI	0.675*	0.769*	0.755*	0.053	0.537
	ODI	0.516	0.264	0.090	0.401	0.456
Right	FA	0.344	0.800*	0.758*	0.509	0.631*
	MD	− 0.582*	− 0.822*	− 0.901*	− 0.633*	− 0.756*
	NDI	0.563*	0.794*	0.855*	− 0.079	0.482
	ODI	0.589*	0.282	0.449	0.198	0.449

*Significant *p* < 0.00125 (0.05/40)

### Correlation between DTI and NODDI metrics in each tract

Please refer to Online Resources 3–7.

### Association of FA with MD, NDI and ODI

FA was significantly negatively correlated with MD and significantly positively correlated with NDI for the left and right side in all the tracts except the ILF. There were significant negative correlations between FA and MD in the left and right ILF but no significant correlations between FA and NDI. There were no significant correlations between FA and ODI in any of the tracts.

### Association of MD with NDI and ODI

MD was significantly negatively correlated with NDI in all tracts bilaterally, except for the ILF where there was only a significant negative correlation between the left ILF MD vs left ILF NDI. For ODI and MD, there were significant negative correlations bilaterally in the cingulum, ILF and optic radiations, but in the CST, only the left MD was significantly negatively correlated with left ODI, and there were no significant correlations in the fornix.

### Multiple regression model

The multiple regression model assessed the impact of different variables (gestational age, PMA at scan, gender, type of tract, side of the tract) on diffusion measures (Online Resources Tables 8-11). FA was not normally distributed on the Shapiro-Wilk test or Q-Q plot, so outliers were removed and data transformed using 1/square transformation (*n* = 309 in the base analysis and *n* = 295 after the transform and outlier removal).

Increasing PMA at scan significantly influenced all the diffusion measures: FA (*p* = 0.000), MD (*p* = 0.009), NDI (*p* = 0.012) and ODI (*p* = 0.008). GA also significantly influenced FA (*p* = 0.000), NDI (*p* = 0.000) and MD (*p* = 0.002) but not ODI (*p* = 0.442). The interaction between GA × PMA at scan significantly influenced FA (*p* = 0.000), MD (*p* = 0.002) and NDI (*p* = 0.000) but not ODI (*p* = 0.269).

Gender influenced NDI, as a main effect (*p* = 0.014) and through interacting with PMA at scan (*p* = 0.009), but it did not affect any of the other diffusion measures. Side did not significantly affect any diffusion measures as a main effect (FA *p* = 0.829, MD *p* = 0.717, NDI *p* = 0.829 and ODI *p* = 0.607) or through interacting with PMA (FA *p* = 0.772, MD *p* = 0.669, NDI *p* = 0.838 and ODI *p* = 0.626).

The rate of change of the diffusion measures with increasing PMA significantly differed between tracts. The rate of change in the cingulum was used as a baseline and compared with the other tracts. The rate of change significantly differed between the cingulum and the CST for FA (*p* = 0.006); MD (*p* = 0.004) and NDI (*p* = 0.022); fornix for MD (*p* = 0.040), NDI (*p* = 0.041) and ODI (*p* = 0.011) and ILF for NDI (*p* = 0.001). Post hoc tests provide further information about which tracts differ from each other (Online Resources 8-11).

The multiple regression model predicted the following variances for each of the diffusion measures: FA *r*^2^ = 0.6706, MD *r*^2^ = 0.7485, NDI *r*^2^ = 0.7990, ODI *r*^2^ = 0.5106.

### Regional changes in white matter microstructure within tracts

**Fig. 2:**
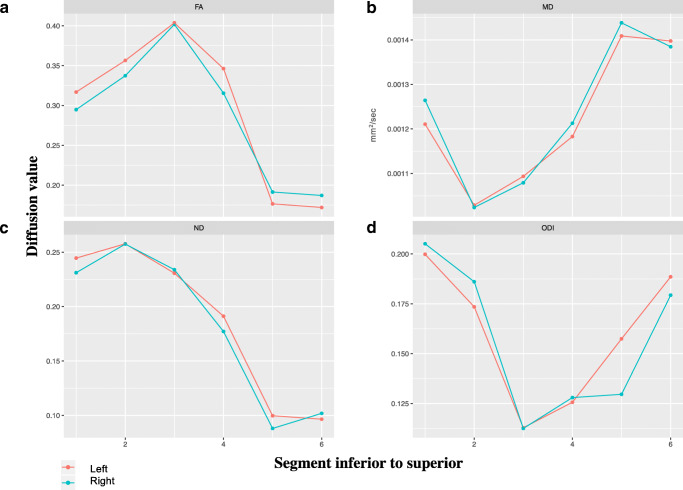
Average diffusion measures FA (**a**), MD (**b**), NDI (**c**) and ODI (**d**) from inferior (segment 1) to superior (segment 6) in the corticospinal tracts

**Fig. 3:**
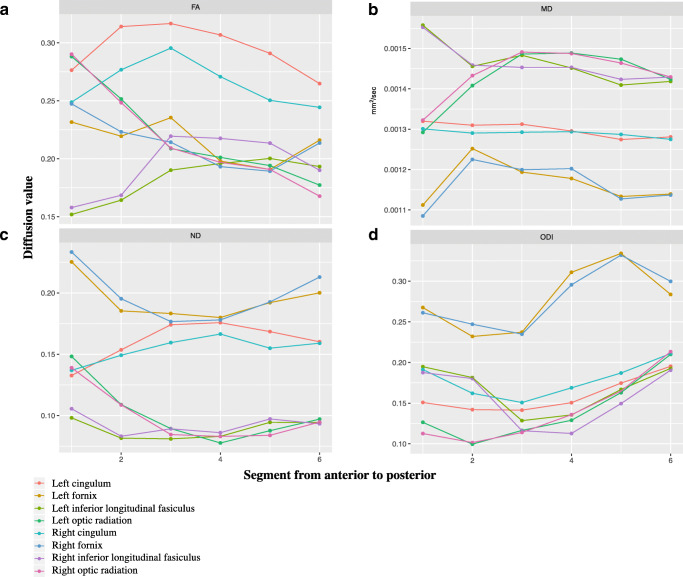
Average diffusion measures FA (**a**), MD (**b**), NDI (**c**) and ODI (**d**) from anterior (segment 1) to posterior (segment 6) in the cingulum, fornix, inferior longitudinal fasciculus and optic radiations

Asynchronous development of WM microstructure was observed within all WM tracts (Figs. 2a–d and 3a–d). The CST had the region with the greatest FA and NDI and lowest ODI and MD compared with the other tracts, and this was present in the most central part of the tract. The cingulum also had a high FA and NDI and low ODI at the centre of the tract, whilst MD remained similar along the tract. Within the fornix, the greatest FA and NDI and lowest MD and ODI were anterior and posteriorly. The ILF had the greatest FA and lowest ODI at the centre of the tract, MD was greatest anteriorly and NDI remained low throughout. The OR had the greatest FA and NDI and lowest ODI and MD anteriorly.

## Discussion

We investigated the microstructural maturation of specific WM tracts in preterm neonates born between 24^+2^ and 36^+4^ weeks GA and scanned between 25^+3^ and 43^+1^ weeks PMA who had normal neurodevelopmental outcomes at 2 years of age. Using a combination of NODDI and DTI metrics, we provided a more specific interpretation of the diffusion data compared with studies using DTI metrics alone. Unlike most other studies, we extracted these diffusion metrics at cross-sectional regions along delineated tracts, as well as for the whole tract, to elucidate regional patterns of WM maturation.

### Using DTI and NODDI to interpret WM microstructural maturation

WM maturation is associated with increasing axonal organisation, pre-myelination and myelination, which progressively restricts water diffusion perpendicular to the direction of the axonal fibre, as represented in our data and previous studies by increasing FA and decreasing MD [41, 42]. Zhang et al. described how changes in FA can be explained by varying combinations of NDI and ODI in adults [26]. In our neonatal cohort, we found that increasing FA was strongly correlated with increasing NDI rather than decreasing ODI. This suggests that increasing FA in WM tracts during this preterm to term period is mainly attributed to increasing axonal growth/density/packing/diameter or pre-myelination/myelination changes, rather than changes in axon coherence or geometry. WM NDI has previously been reported to increase with age in neonates [29], children [43, 44] and adolescents [45], demonstrating that WM tracts undergo these changes beyond the neonatal period and throughout childhood. Greater NDI in childhood has been associated with better neurodevelopmental outcomes, IQ [18, 31], visual motor integration [18], motor/behavioural/emotional scores [31], language [20] and maths [46].

With the exception of the right cingulum, ODI did not correlate with PMA at scan in any tract, and there were no significant correlations between ODI and FA. Other studies investigating normal WM development in childhood and adolescence report no [38, 43, 47] or very weak [44] association between ODI and age. As far as we are aware, there have not been any other studies using NODDI to investigate normal WM development in healthy preterm infants with normal neurodevelopmental outcomes. However, follow-up studies report reduced FA and greater ODI may be associated with poorer neurodevelopmental outcomes including IQ [18], motor/emotional/behavioural scores [31] and language [20].

### Comparing WM maturation between and within tracts

The fornix matures earlier than the other tracts, as demonstrated by its high FA, high NDI and low MD. This finding is supported by post-mortem studies, which report the fornix as one of the first tracts to emerge and is evident by 12 weeks GA [48, 49], and that by 19–24 weeks GA, it is well developed, with entire trajectories visible on post-mortem foetal brain samples using DTI at 19 weeks GA [50]. Using in vivo foetal DTI tractography, the fornix can be depicted as early as 13 weeks gestation [51], and by 1–4 months postnatal age, it is one of the most mature WM tracts, only superseded by the CST [52].

Rapid maturation [53] and early myelination in the CST have been reported on histological [54] and DTI studies [55–57]. Our findings corroborate these reports, with the CST displaying the most rapid increase in FA and NDI with increasing PMA. In addition, we found a strong positive correlation between FA and NDI in the left (*r* = 0.921) and right CST (*r* = 0.944) and between NDI and PMA at scan in the left (*r* = 0.796) and right (*r* = 0.794) CST. Our within-tract analysis of the CST demonstrated the greatest increase in FA and NDI, with a corresponding reduction in MD and ODI, at the level of the internal capsule. Early maturation and myelination within the CST at the level of the internal capsule have been reported in DTI [57, 58] and histological studies [59].

In contrast, the ILF is the least mature tract, based on dMRI metrics, with the lowest FA and greatest MD. Association fibres are among the last WM tracts to develop [38, 50, 57, 60, 61], are only visible on foetal DTI tractography at 19 weeks gestation [50, 51, 62] and have the lowest FA through the foetal life and early infancy [61]. Low FA during this preterm to term period was associated with a relatively low and stable NDI, suggesting that the ILF has low neurite density and myelination compared with the other tracts. This is consistent with other studies that report that myelination in the ILF does not commence until the third postnatal month [58, 63] but then rapidly increases during the first year of life [64] and is not fully mature until the third decade [8, 65]. There was inhomogeneous WM development within the ILF, which has previously been reported in adults [33]. FA was greatest in the centre of the ILF coinciding with a reduction in ODI but no change in NDI, implying that changes in FA are predominantly driven by axonal cohesion.

In the cingulum, FA increased from the anterior cingulate gyrus towards the midsection, and this coincided with a rise in NDI and slight reduction in ODI, suggesting the midsection has the greatest neurite density, myelination and least dispersion. MD remained stable along the tract. Assessing the tractography images visually, the fibres were most cohesive in the centre but dispersed in the anterior and particularly posterior pole, consistent with the ODI results. Another DTI study in adults reported regional changes in WM maturation along the cingulum, supporting our findings that maturation is not uniform along the tract [33].

The optic radiations had the greatest FA and NDI and lowest MD in the most anterior part of the tract, corresponding to the lateral geniculate nucleus of the thalamus, whilst ODI was greatest at the most posterior part of the tract at the level of the visual cortex. Again, suggesting that maturation of the tract proceeds from central to peripheral regions of the brain [58].

### Impact of other variables on WM maturation

Our model provided further information about the influence of different covariates on diffusion measures. Our model did not reveal a significant effect of lateralisation on diffusion measures. Visually inspecting the linear plots in Online Resources 2a-d, the only noticeable difference appeared to be in FA between the left and right cingulum, a finding previously reported as significantly different in infants [66, 67] and adults [68, 69]. Other studies have reported lateralisation in some but not all WM pathways in foetuses [70], infants [15, 66, 67, 71] and children [72], but results are inconsistent. A systematic review investigating how DTI metrics vary in preterm neonates scanned before term-equivalent age concluded that lateralisation may occur, but the evidence is conflicting and further work is required [15].

Gender influenced NDI but not FA, MD or ODI in our preterm cohort. Other studies have conflicting results regarding the impact of gender on diffusion measures, both in preterm infants [15, 73] and older subjects [43–45, 64, 72, 74]. GA had a significant effect on all our diffusion measures except ODI, as a main effect and through interactions with PMA at scan. Lower GA has previously been identified as a risk factor for diffuse WM injury in preterm infants [73], but results are conflicting [15].

### Limitations

This study used cross-sectional data to investigate age-related differences in WM microstructure, whereas longitudinal data could provide more accurate data regarding changes over time. In addition, our study population was small, as we were investigating normal WM maturation in a population of preterm neonates with normal neurodevelopmental outcomes at 2 years. Whilst informative, our model only explains a part of the variation in the diffusion measures, and there are other covariates that we have not accounted for.

## Conclusion

WM maturation from the preterm to term period is associated with increasing FA and NDI and decreasing MD, as WM tracts become increasingly organised, myelinated and increase in density. WM maturation does not occur uniformly between WM tracts, with projection tracts maturing more rapidly than association tracts. Regional differences occur within WM tracts, with the most mature regions occurring centrally rather than peripherally (cingulum, OR) and inferiorly rather than superiorly (CST). Future studies should consider using NODDI or the recent extension to NODDI (multi-TE NODDI [75]) to provide a more in-depth interpretation of the DTI metrics.

## Supplementary Information

ESM 1 (DOCX 474 kb)

## Data Availability

Not applicable.

## References

[1] Goldenberg RL, Culhane JF, Iams JD, Romero R (2008). Epidemiology and causes of preterm birth. Lancet.

[2] Bhutta AT, Cleves MA, Casey PH, Cradock MM, Anand KJ (2002). Cognitive and behavioral outcomes of school-aged children who were born preterm: a meta-analysis. Jama.

[3] Marlow N, Wolke D, Bracewell MA, Samara M (2005). Neurologic and developmental disability at six years of age after extremely preterm birth. N Engl J Med.

[4] Volpe JJ (2003). Cerebral white matter injury of the premature infant-more common than you think. Pediatrics.

[5] Counsell SJ, Allsop JM, Harrison MC, Larkman DJ, Kennea NL, Kapellou O, Cowan FM, Hajnal JV, Edwards AD, Rutherford MA (2003). Diffusion-weighted imaging of the brain in preterm infants with focal and diffuse white matter abnormality. Pediatrics.

[6] Dyet LE, Kennea N, Counsell SJ, Maalouf EF, Ajayi-Obe M, Duggan PJ, Harrison M, Allsop JM, Hajnal J, Herlihy AH, Edwards B, Laroche S, Cowan FM, Rutherford MA, Edwards AD (2006). Natural history of brain lesions in extremely preterm infants studied with serial magnetic resonance imaging from birth and neurodevelopmental assessment. Pediatrics.

[7] Counsell SJ, Edwards AD, Chew AT, Anjari M, Dyet LE, Srinivasan L, Boardman JP, Allsop JM, Hajnal JV, Rutherford MA, Cowan FM (2008). Specific relations between neurodevelopmental abilities and white matter microstructure in children born preterm. Brain J Neurol.

[8] Lebel C, Deoni S (2018). The development of brain white matter microstructure. Neuroimage.

[9] Barkovich AJ, Lyon G, Evrard P (1992). Formation, maturation, and disorders of white matter. AJNR Am J Neuroradiol.

[10] Poduslo SE, Jang Y (1984). Myelin development in infant brain. Neurochem Res.

[11] Baumann N, Pham-Dinh D (2001). Biology of oligodendrocyte and myelin in the mammalian central nervous system. Physiol Rev.

[12] Rogers CE, Smyser T, Smyser CD, Shimony J, Inder TE, Neil JJ (2016). Regional white matter development in very preterm infants: perinatal predictors and early developmental outcomes. Pediatr Res.

[13] Aeby A, Liu Y, De Tiege X, Denolin V, David P, Baleriaux D, Kavec M, Metens T, Van Bogaert P (2009). Maturation of thalamic radiations between 34 and 41 weeks’ gestation: a combined voxel-based study and probabilistic tractography with diffusion tensor imaging. AJNR Am J Neuroradiol.

[14] Berman JI, Mukherjee P, Partridge SC, Miller SP, Ferriero DM, Barkovich AJ, Vigneron DB, Henry RG (2005). Quantitative diffusion tensor MRI fiber tractography of sensorimotor white matter development in premature infants. NeuroImage.

[15] Pannek K, Scheck SM, Colditz PB, Boyd RN, Rose SE (2014). Magnetic resonance diffusion tractography of the preterm infant brain: a systematic review. Dev Med Child Neurol.

[16] Hasegawa T, Yamada K, Morimoto M, Morioka S, Tozawa T, Isoda K, Murakami A, Chiyonobu T, Tokuda S, Nishimura A, Nishimura T, Hosoi H (2011). Development of corpus callosum in preterm infants is affected by the prematurity: in vivo assessment of diffusion tensor imaging at term-equivalent age. Pediatr Res.

[17] Thompson DK, Lee KJ, Egan GF, Warfield SK, Doyle LW, Anderson PJ, Inder TE (2014). Regional white matter microstructure in very preterm infants: predictors and 7 year outcomes. Cortex.

[18] Young JM, Vandewouw MM, Mossad SI, Morgan BR, Lee W, Smith ML, Sled JG, Taylor MJ (2019). White matter microstructural differences identified using multi-shell diffusion imaging in six-year-old children born very preterm. Neuroimage Clin.

[19] van Kooij BJ, de Vries LS, Ball G, van Haastert IC, Benders MJ, Groenendaal F, Counsell SJ (2012). Neonatal tract-based spatial statistics findings and outcome in preterm infants. AJNR Am J Neuroradiol.

[20] Murner-Lavanchy IM, Kelly CE, Reidy N, Doyle LW, Lee KJ, Inder T, Thompson DK, Morgan AT, Anderson PJ (2018). White matter microstructure is associated with language in children born very preterm. Neuroimage Clin.

[21] Kelly CE, Thompson DK, Spittle AJ, Chen J, Seal ML, Anderson PJ, Doyle LW, Cheong JL (2020) Regional brain volumes, microstructure and neurodevelopment in moderate-late preterm children. Arch Dis Child Fetal Neonatal Ed. 10.1136/archdischild-2019-317941 10.1136/archdischild-2019-317941 32132139

[22] Cheng Y, Chou KH, Chen IY, Fan YT, Decety J, Lin CP (2010). Atypical development of white matter microstructure in adolescents with autism spectrum disorders. Neuroimage.

[23] Skranes J, Vangberg TR, Kulseng S, Indredavik MS, Evensen KA, Martinussen M, Dale AM, Haraldseth O, Brubakk AM (2007). Clinical findings and white matter abnormalities seen on diffusion tensor imaging in adolescents with very low birth weight. Brain J Neurol.

[24] Tournier JD, Calamante F, Connelly A (2012). MRtrix: diffusion tractography in crossing fiber regions. Int J Imaging Syst Technol.

[25] Jones DK, Knosche TR, Turner R (2013). White matter integrity, fiber count, and other fallacies: the do's and don'ts of diffusion MRI. Neuroimage.

[26] Zhang H, Schneider T, Wheeler-Kingshott CA, Alexander DC (2012). NODDI: practical in vivo neurite orientation dispersion and density imaging of the human brain. Neuroimage.

[27] Jespersen SN, Leigland LA, Cornea A, Kroenke CD (2012). Determination of axonal and dendritic orientation distributions within the developing cerebral cortex by diffusion tensor imaging. IEEE Trans Med Imaging.

[28] Sepehrband F, Clark KA, Ullmann JFP, Kurniawan ND, Leanage G, Reutens DC, Yang Z (2015). Brain tissue compartment density estimated using diffusion-weighted MRI yields tissue parameters consistent with histology. Hum Brain Mapp.

[29] Batalle D, Hughes EJ, Zhang H, Tournier JD, Tusor N, Aljabar P, Wali L, Alexander DC, Hajnal JV, Nosarti C, Edwards AD, Counsell SJ (2017). Early development of structural networks and the impact of prematurity on brain connectivity. Neuroimage.

[30] Kunz N, Zhang H, Vasung L, O'Brien KR, Assaf Y, Lazeyras F, Alexander DC, Hüppi PS (2014). Assessing white matter microstructure of the newborn with multi-shell diffusion MRI and biophysical compartment models. NeuroImage.

[31] Kelly CE, Thompson DK, Chen J, Leemans A, Adamson CL, Inder TE, Cheong JLY, Doyle LW, Anderson PJ (2016). Axon density and axon orientation dispersion in children born preterm. Hum Brain Mapp.

[32] Groeschel S, Tournier JD, Northam GB, Baldeweg T, Wyatt J, Vollmer B, Connelly A (2014). Identification and interpretation of microstructural abnormalities in motor pathways in adolescents born preterm. Neuroimage.

[33] Martensson J, Latt J, Ahs F, Fredrikson M, Soderlund H, Schioth HB, Kok J, Kremer B, van Westen D, Larsson EM, Nilsson M (2018). Diffusion tensor imaging and tractography of the white matter in normal aging: the rate-of-change differs between segments within tracts. Magn Reson Imaging.

[34] Lebel C, Caverhill-Godkewitsch S, Beaulieu C (2010). Age-related regional variations of the corpus callosum identified by diffusion tensor tractography. NeuroImage.

[35] Goodlett CB, Fletcher PT, Gilmore JH, Gerig G (2009). Group analysis of DTI fiber tract statistics with application to neurodevelopment. NeuroImage.

[36] Johnson RT, Yeatman JD, Wandell BA, Buonocore MH, Amaral DG, Nordahl CW (2014). Diffusion properties of major white matter tracts in young, typically developing children. NeuroImage.

[37] Chen Z, Zhang H, Yushkevich PA, Liu M, Beaulieu C (2016). Maturation along white matter tracts in human brain using a diffusion tensor surface model tract-specific analysis. Front Neuroanat.

[38] Lynch KM, Cabeen RP, Toga AW, Clark KA (2020). Magnitude and timing of major white matter tract maturation from infancy through adolescence with NODDI. Neuroimage.

[39] Tristan-Vega A, Arribas JI (2007) A fast B-spline pseudo-inversion algorithm for consistent image registration. Proceedings of the International Conference on Computer Analysis Images and Patterns (CAIP), Vienna, Austria:768–775

[40] Makropoulos A, Gousias IS, Ledig C, Aljabar P, Serag A, Hajnal JV, Edwards AD, Counsell SJ, Rueckert D (2014). Automatic whole brain MRI segmentation of the developing neonatal brain. IEEE Trans Med Imaging.

[41] Huppi PS, Warfield S, Kikinis R, Barnes PD, Zientara GP, Jolesz FA, Tsuji MK, Volpe JJ (1998). Quantitative magnetic resonance imaging of brain development in premature and mature newborns. Ann Neurol.

[42] Anblagan D, Bastin ME, Sparrow S, Piyasena C, Pataky R, Moore EJ, Serag A, Wilkinson AG, Clayden JD, Semple SI, Boardman JP (2015). Tract shape modeling detects changes associated with preterm birth and neuroprotective treatment effects. Neuroimage Clin.

[43] Mah A, Geeraert B, Lebel C (2017). Detailing neuroanatomical development in late childhood and early adolescence using NODDI. PLoS One.

[44] Genc S, Malpas CB, Holland SK, Beare R, Silk TJ (2017). Neurite density index is sensitive to age related differences in the developing brain. Neuroimage.

[45] Geeraert BL, Lebel RM, Lebel C (2019). A multiparametric analysis of white matter maturation during late childhood and adolescence. Hum Brain Mapp.

[46] Collins SE, Spencer-Smith M, Murner-Lavanchy I, Kelly CE, Pyman P, Pascoe L, Cheong J, Doyle LW, Thompson DK, Anderson PJ (2019). White matter microstructure correlates with mathematics but not word reading performance in 13-year-old children born very preterm and full-term. Neuroimage Clin.

[47] Chang YS, Owen JP, Pojman NJ, Thieu T, Bukshpun P, Wakahiro MLJ, Berman JI, Roberts TPL, Nagarajan SS, Sherr EH, Mukherjee P (2015). White matter changes of neurite density and fiber orientation dispersion during human brain maturation. PLoS One.

[48] Radoš M, Judaš M, Kostović I (2006). In vitro MRI of brain development. Eur J Radiol.

[49] Judas M, Rados M, Jovanov-Milosevic N, Hrabac P, Stern-Padovan R, Kostovic I (2005). Structural, immunocytochemical, and mr imaging properties of periventricular crossroads of growing cortical pathways in preterm infants. AJNR Am J Neuroradiol.

[50] Huang H, Zhang J, Wakana S, Zhang W, Ren T, Richards LJ, Yarowsky P, Donohue P, Graham E, van Zijl PCM, Mori S (2006). White and gray matter development in human fetal, newborn and pediatric brains. NeuroImage.

[51] Huang H, Xue R, Zhang J, Ren T, Richards LJ, Yarowsky P, Miller MI, Mori S (2009). Anatomical characterization of human fetal brain development with diffusion tensor magnetic resonance imaging. J Neurosci.

[52] Dubois J, Dehaene-Lambertz G, Perrin M, Mangin J, Cointepas Y, Duchesnay E, Le Bihan D, Hertz-Pannier L (2008). Asynchrony of the early maturation of white matter bundles in healthy infants: quantitative landmarks revealed noninvasively by diffusion tensor imaging. Hum Brain Mapp.

[53] Dimond D, Rohr CS, Smith RE, Dhollander T, Cho I, Lebel C, Dewey D, Connelly A, Bray S (2020). Early childhood development of white matter fiber density and morphology. Neuroimage.

[54] Gilles FH, Shankle W, Dooling EC (1983) Myelinated tracts: growth patterns. In: The Developing Human Brain. Butterworth-Heinemann, pp. 117–183. 10.1016/B978-0-7236-7017-9.50018-1

[55] Zanin E, Ranjeva JP, Confort-Gouny S, Guye M, Denis D, Cozzone PJ, Girard N (2011). White matter maturation of normal human fetal brain. An in vivo diffusion tensor tractography study. Brain Behav.

[56] Qiu A, Mori S, Miller MI (2015). Diffusion tensor imaging for understanding brain development in early life. Annu Rev Psychol.

[57] Kulikova S, Hertz-Pannier L, Dehaene-Lambertz G, Buzmakov A, Poupon C, Dubois J (2015). Multi-parametric evaluation of the white matter maturation. Brain Struct Funct.

[58] Dubois J, Dehaene-Lambertz G, Kulikova S, Poupon C, Huppi PS, Hertz-Pannier L (2014). The early development of brain white matter: a review of imaging studies in fetuses, newborns and infants. Neuroscience.

[59] Gilles FH, Shankle W, Dooling EC (1983) The developing human brain: growth and epidemiologic neuropathology. Butterworth-Heinemann. 10.1016/B978-0-7236-7017-9.50018-1

[60] Kinney HC, Brody BA, Kloman AS, Gilles FH (1988). Sequence of central nervous system myelination in human infancy. II. Patterns of myelination in autopsied infants. J Neuropathol Exp Neurol.

[61] Ouyang M, Dubois J, Yu Q, Mukherjee P, Huang H (2019). Delineation of early brain development from fetuses to infants with diffusion MRI and beyond. NeuroImage.

[62] Mitter C, Prayer D, Brugger PC, Weber M, Kasprian G (2015). In vivo tractography of fetal association fibers. PLoS One.

[63] Hasegawa M, Houdou S, Mito T, Takashima S, Asanuma K, Ohno T (1992). Development of myelination in the human fetal and infant cerebrum: a myelin basic protein immunohistochemical study. Brain Dev.

[64] Geng X, Gouttard S, Sharma A, Gu H, Styner M, Lin W, Gerig G, Gilmore JH (2012). Quantitative tract-based white matter development from birth to age 2years. NeuroImage.

[65] Lebel C, Beaulieu C (2011). Longitudinal development of human brain wiring continues from childhood into adulthood. J Neurosci.

[66] Dean DC, Planalp EM, Wooten W, Adluru N, Kecskemeti SR, Frye C, Schmidt CK, Schmidt NL, Styner MA, Goldsmith HH, Davidson RJ, Alexander AL (2017). Mapping white matter microstructure in the one month human brain. Sci Rep.

[67] Cohen AH, Wang R, Wilkinson M, MacDonald P, Lim AR, Takahashi E (2016). Development of human white matter fiber pathways: from newborn to adult ages. Int J Dev Neurosci.

[68] Takao H, Hayashi N, Ohtomo K (2011). White matter asymmetry in healthy individuals: a diffusion tensor imaging study using tract-based spatial statistics. Neuroscience.

[69] Yin X, Han Y, Ge H, Xu W, Huang R, Zhang D, Xu J, Fan L, Pang Z, Liu S (2013). Inferior frontal white matter asymmetry correlates with executive control of attention. Hum Brain Mapp.

[70] Song JW, Mitchell PD, Kolasinski J, Ellen Grant P, Galaburda AM, Takahashi E (2015). Asymmetry of white matter pathways in developing human brains. Cereb Cortex.

[71] Dubois J, Hertz-Pannier L, Cachia A, Mangin JF, Le Bihan D, Dehaene-Lambertz G (2009). Structural asymmetries in the infant language and sensori-motor networks. Cereb Cortex.

[72] Lebel C, Walker L, Leemans A, Phillips L, Beaulieu C (2008). Microstructural maturation of the human brain from childhood to adulthood. NeuroImage.

[73] Barnett ML, Tusor N, Ball G, Chew A, Falconer S, Aljabar P, Kimpton JA, Kennea N, Rutherford M, David Edwards A, Counsell SJ (2018). Exploring the multiple-hit hypothesis of preterm white matter damage using diffusion MRI. Neuroimage Clin.

[74] Kodiweera C, Alexander AL, Harezlak J, McAllister TW, Wu Y-C (2016). Age effects and sex differences in human brain white matter of young to middle-aged adults: a DTI, NODDI, and q-space study. NeuroImage.

[75] Gong T, Tong Q, He H, Sun Y, Zhong J, Zhang H (2020). MTE-NODDI: multi-TE NODDI for disentangling non-T2-weighted signal fractions from compartment-specific T2 relaxation times. Neuroimage.

