# Supplementary material for: Diffusion magnetic resonance imaging assessment of regional white matter maturation in preterm neonates
Source: Neuroradiology. 2020 Oct 29;63(4):573–83. doi: 10.1007/s00234-020-02584-9 (PMC7966229; doi:10.1007/s00234-020-02584-9)
Supplement: Supplementary file 1 — (DOCX 474 kb) [file 234_2020_2584_MOESM1_ESM.docx]

**SUPPLEMENTARY FILES:**

**Online Resource 1** Regions included and excluded to obtain white matter tracts

| **Tract** | **Regions included** | **Regions excluded** |
| --- | --- | --- |
| Cingulum | Anterior cingulate gyrus, posterior cingulate gyrus | Amygdala, parahippocampal gyrus, hippocampus, pallidum, putamen, thalamus, caudate |
| Corticospinal tract | Manually drawn cerebral peduncle and posterior limb of the internal capsule mask, precentral gyrus | Manually drawn interhemispheric mask, postcentral gyrus mask |
| Fornix | Hippocampus, manually drawn region of interest to represent the fornix column | Precentral gyrus, middle cingulate gyrus, posterior cingulate gyrus, calcarine cortex, superior occipital gyrus, fusiform gyrus, caudate, pallidum, middle temporal pole, superior temporal pole, putamen, a manually drawn mask to represent the mesencephalon |
| Inferior longitudinal fasciculus | Middle temporal pole, superior temporal pole, inferior occipital gyrus | Amygdala, parahippocampal gyrus |
| Optic radiations | Thalamus, inferior, superior and middle occipital gyrus | Manually drawn interhemispheric mask, inferior parietal gyrus, inferior, superior and middle temporal gyrus |

**Online Resources** **2a-d** Linear plots demonstrating the association between the diffusion measures FA (a), MD (b), NDI (c) and ODI (d) with post-menstrual age at scan in white matter tracts

2a)

2b)

2c)

2d)

**Online Resource 3** Correlation analysis between diffusion measures in the cingulum

|  | | Left cingulum | | | | Right cingulum | | | |
| --- | --- | --- | --- | --- | --- | --- | --- | --- | --- |
|  |  | FA | MD | NDI | ODI | FA | MD | NDI | ODI |
| Left cingulum | FA | 1 |  |  |  |  |  |  |  |
|  | MD | **-0.704*** | 1 |  |  |  |  |  |  |
|  | NDI | **0.868*** | **-0.813*** | 1 |  |  |  |  |  |
|  | ODI | 0.102 | **-0.691*** | 0.442 | 1 |  |  |  |  |
| Right cingulum | FA | **0.834*** | **-0.583*** | **0.675*** | 0.121 | 1 |  |  |  |
|  | MD | **-0.709*** | **0.887*** | **-0.815*** | **-0.602*** | **-0.671*** | 1 |  |  |
|  | NDI | **0.699*** | **-0.817*** | **0.813*** | **0.586*** | **0.754*** | **-0.867*** | 1 |  |
|  | ODI | 0.302 | **-0.737*** | **0.585*** | **0.850*** | 0.221 | **-0.798*** | **0.719*** | 1 |

**Online Resource 4** Correlation analysis between diffusion measures in the CST

|  | | Left CST | | | | Right CST | | | |
| --- | --- | --- | --- | --- | --- | --- | --- | --- | --- |
|  |  | FA | MD | NDI | ODI | FA | MD | NDI | ODI |
| Left CST | FA | 1 |  |  |  |  |  |  |  |
|  | MD | **-0.805*** | 1 |  |  |  |  |  |  |
|  | NDI | **0.921*** | **-0.949*** | 1 |  |  |  |  |  |
|  | ODI | -0.007 | **-0.556*** | 0.338 | 1 |  |  |  |  |
| Right CST | FA | **0.931*** | **-0.818*** | **0.908*** | 0.087 | 1 |  |  |  |
|  | MD | **-0.755*** | **0.961*** | **-0.895*** | -0.541 | **-0.833*** | 1 |  |  |
|  | NDI | **0.886*** | **-0.925*** | **0.954*** | 0.323 | **0.944*** | **-0.944*** | 1 |  |
|  | ODI | 0.007 | -0.510 | 0.268 | **0.846*** | -0.002 | -0.528 | 0.283 | 1 |

**Online Resource 5** Correlation analysis between diffusion measures in the fornix

|  | | Left fornix | | | | Right fornix | | | |
| --- | --- | --- | --- | --- | --- | --- | --- | --- | --- |
|  |  | FA | MD | NDI | ODI | FA | MD | NDI | ODI |
| Left fornix | FA | 1 |  |  |  |  |  |  |  |
|  | MD | **-0.785*** | 1 |  |  |  |  |  |  |
|  | NDI | **0.726*** | **-0.838*** | 1 |  |  |  |  |  |
|  | ODI | -0.121 | -0.423 | 0.432 | 1 |  |  |  |  |
| Right fornix | FA | **0.830*** | **-0.672*** | **0.722*** | -0.043 | 1 |  |  |  |
|  | MD | **-0.794*** | **0.797*** | **-0.803*** | -0.122 | **-0.797*** | 1 |  |  |
|  | NDI | **0.750*** | **-0.757*** | **0.876*** | 0.206 | **0.864*** | **-0.894*** | 1 |  |
|  | ODI | 0.172 | -0.434 | 0.464 | 0.559* | 0.071 | -0.532 | 0.460 | 1 |

**Online Resource 6** Correlation analysis between diffusion measures in the ILF

|  | | Left ILF | | | | Right ILF | | | |
| --- | --- | --- | --- | --- | --- | --- | --- | --- | --- |
|  |  | FA | MD | NDI | ODI | FA | MD | NDI | ODI |
| Left ILF | FA | 1 |  |  |  |  |  |  |  |
|  | MD | **-0.651*** | 1 |  |  |  |  |  |  |
|  | NDI | 0.552 | **-0.633*** | 1 |  |  |  |  |  |
|  | ODI | 0.334 | **-0.837*** | **0.689*** | 1 |  |  |  |  |
| Right ILF | FA | **0.854*** | **-0.582*** | 0.324 | 0.268 | 1 |  |  |  |
|  | MD | **-0.634*** | **0.897*** | **-0.587*** | **-0.730*** | -0.549 | 1 |  |  |
|  | NDI | 0.372 | -0.517 | **0.752*** | 0.523 | 0.190 | -0.526 | 1 |  |
|  | ODI | 0.106 | **-0.618*** | 0.529 | **0.787*** | -0.084 | **-0.679*** | **0.674*** | 1 |

**Online Resource 7** Correlation analysis between diffusion measures in the optic radiations

|  | | Left optic radiation | | | | Right optic radiation | | | |
| --- | --- | --- | --- | --- | --- | --- | --- | --- | --- |
|  |  | FA | MD | NDI | ODI | FA | MD | NDI | ODI |
| Left optic radiation | FA | 1 |  |  |  |  |  |  |  |
|  | MD | **-0.712*** | 1 |  |  |  |  |  |  |
|  | NDI | **0.733*** | **-0.831*** | 1 |  |  |  |  |  |
|  | ODI | -0.003 | **-0.652*** | 0.466 | 1 |  |  |  |  |
| Right optic radiation | FA | **0.888*** | **-0.632*** | **0.658*** | 0.070 | 1 |  |  |  |
|  | MD | **-0.617*** | **0.940*** | **-0.789*** | **-0.698*** | **-0.614*** | 1 |  |  |
|  | NDI | **0.672*** | **-0.792*** | **0.897*** | 0.480 | **0.656*** | **-0.824*** | 1 |  |
|  | ODI | 0.178 | **-0.747*** | **0.574*** | **0.904*** | 0.113 | **-0.805*** | **0.540*** | 1 |

**Online Resource 8** Multiple regression results for the influence different variables on mean diffusivity

| Variables included in the model | Observed coefficient | Lower confidence interval 95% | Upper confidence interval 95% | p-value  (3 decimal places) |
| --- | --- | --- | --- | --- |
| Female | 1.1 x 10^-5^ | -1.1 x 10^-4^ | 1.4 x 10^-4^ | p=0.867 |
| Gestational age | 2.1 x 10^-4^ | 7.6 x 10^-5^ | 3.5 x 10^-4^ | **p=0.002** |
| PMA at scan | 7.7 x 10^-6^ | -1.4 x 10^-5^ | 2.2 x 10^-6^ | **p=0.009** |
| Side | -2.2 x 10^-5^ | -1.3 x 10^-4^ | -1.2 x 10^-4^ | p=0.717 |
| Tract:  CST  Fornix  ILF OR | 2.4 x 10^-4^  4.9 x 10^-5^  9.0 x 10^-5^  1.4 x 10^-4^ | 4.1 x 10^-5^  -1.5 x 10^-4^  -1.3 x 10^-4^  -8.7 x 10^-5^ | 4.3 x 10^-4^  2.3 x 10^-4^  3.2 x 10^-4^  3.3 x 10^-4^ | **p=0.019**  p=0.610  p=0.431  p=0.203 |
| Gender x PMA | -4.5 x 10^-7^ | -4.2 x 10^-6^ | 3.1 x 10^-6^ | p=0.805 |
| Side x PMA | 7.2 x 10^-7^ | -2.7 x 10^-6^ | 4.0 x 10^-6^ | p=0.669 |
| PMA x GA | -6.0 x 10^-6^ | -9.9 x 10^-6^ | -2.2 x 10^-6^ | **p=0.002** |
| Tract x PMA:  CST  Fornix  ILF  OR | -8.0 x 10^-6^  -5.4 x 10^-6^  -9.7 x 10^-7^  -4.4 x 10^-6^ | -1.3 x 10^-5^  -1.2 x 10^-5^  -7.3 x 10^-6^  -9.8 x 10^-6^ | 2.6 x 10^-6^  -2.1 x 10^-7^  5.2 x 10^-6^  1.5 x 10^-6^ | **p=0.004**  **p=0.040**  p=0.760  p=0.138 |
| Post-hoc tests for tracts x PMA:  Cingulum vs CST  Cingulum vs fornix  Cingulum vs ILF  Cingulum vs OR  CST vs fornix  CST vs ILF  CST vs OR  Fornix vs ILF  Fornix vs OR  ILF vs OR |  |  |  | **p=0.0045**  **p=0.0401**  p=0.7603  p=0.1382  p=0.1977  **p=0.0107**  p=0.1227  p=0.0949  p=0.6701  p=0.2428 |

* Significant, p<0.05, PMA: postmenstrual age at scan, CST: corticospinal tract, ILF: inferior longitudinal fasciculus, OR: optic radiations. Baseline categorical variables include: gender (male); gestational age (<32/40); side (right); tract (cingulum).

**Online Resource 9** Multiple regression results reporting the influence different variables on neurite density index

| Variables included in the model | Observed coefficient  (4 decimal places) | Lower confidence interval 95%  (4 decimal places) | Upper confidence interval 95%  (4 decimal places) | p-value  (3 decimal places) |
| --- | --- | --- | --- | --- |
| Gender | -0.0507 | -0.0939 | -0.0127 | **p=0.014** |
| Gestational age | -0.0999 | -0.1475 | -0.0592 | **p=0.000** |
| PMA at scan | 0.00233 | 0.0005 | 0.0042 | **p=0.012** |
| Side | -0.0045 | -0.0470 | 0.0344 | p=0.829 |
| Tract:  CST  Fornix  ILF OR | -0.0752  -0.0032  0.1085  0.0461 | -0.1415  -0.0669  0.0391  -0.0232 | -0.0070  0.0680  0.1751  0.1152 | **p=0.028**  p=0.921  **p=0.003**  p=0.178 |
| Gender x PMA | 0.0015 | 0.0004 | 0.0026 | **p=0.009** |
| Side x PMA | 0.0001 | -0.0010 | 0.0013 | p=0.838 |
| PMA x GA | 0.0026 | 0.0015 | 0.0040 | **p=0.000** |
| Tract x PMA:  CST  Fornix  ILF  OR | 0.0022  0.0019  -0.0035  -0.0012 | 0.0003  -0.0002  -0.0056  -0.0030 | 0.0041  0.0035  -0.0017  0.0008 | **p=0.022**  **p=0.041**  **p=0.001**  p=0.222 |
| Post-hoc tests for tracts x PMA:  Cingulum vs CST  Cingulum vs fornix  Cingulum vs ILF  Cingulum vs OR  CST vs fornix  CST vs ILF  CST vs OR  Fornix vs ILF  Fornix vs OR  ILF vs OR |  |  |  | **p=0.022**  **p=0.041**  **p=0.001**  p=0.222  p=0.709  **p=0.000**  **p=0.000**  **p=0.000**  **p=0.000**  **p=0.012** |

* Significant, p<0.05, PMA: postmenstrual age at scan, CST: corticospinal tract, ILF: inferior longitudinal fasciculus, OR: optic radiations. Baseline categorical variables include: gender (male); gestational age (<32/40); side (right); tract (cingulum).

**Online Resource 10** Multiple regression results reporting the influence different variables on orientation dispersion index

| Variables included in the model | Observed coefficient  (4 decimal places) | Lower confidence interval 95%  (4 decimal places) | Upper confidence interval 95%  (4 decimal places) | p-value  (3 decimal places) |
| --- | --- | --- | --- | --- |
| Gender | -0.0219 | -0.0827 | 0.0398 | p=0.490 |
| Gestational age | -0.0268 | -0.0959 | 0.0411 | p=0.442 |
| PMA at scan | 0.0023 | 0.0005 | 0.0039 | **p=0.008** |
| Side | 0.0141 | -0.0413 | 0.0665 | p=0.607 |
| Tract:  CST  Fornix  ILF OR | 0.0605  0.1311  0.0331  0.0124 | -0.0227  0.0658  -0.0587  -0.0777 | 0.1531  0.1845  0.1199  0.0832 | p=0.171  **p=0.000**  p=0.465  p=0.771 |
| Gender x PMA | -0.0219 | -0.0014 | 0.0024 | p=0.582 |
| Side x PMA | -0.0004 | -0.0019 | 0.0012 | p=0.626 |
| PMA x GA | 0.0011 | -0.0009 | 0.0030 | p=0.269 |
| Tract x PMA:  CST  Fornix  ILF  OR | -0.0015  -0.0023  -0.0013  0.0000 | -0.0041  -0.0039  -0.0039  -0.0020 | 0.0008  -0.0005  0.0014  0.0025 | p=0.236  **p=0.011**  p=0.325  p=0.990 |
| Post-hoc tests for tracts x PMA:  Cingulum vs CST  Cingulum vs fornix  Cingulum vs ILF  Cingulum vs OR  CST vs fornix  CST vs ILF  CST vs OR  Fornix vs ILF  Fornix vs OR  ILF vs OR |  |  |  | p=0.2364  **p=0.0108**  p=0.3245  p=0.9903  p=0.4990  p=0.9098  p=0.2943  p=0.4502  **p=0.0396**  p=0.3868 |

* Significant, p<0.05, PMA: postmenstrual age at scan, CST: corticospinal tract, ILF: inferior longitudinal fasciculus, OR: optic radiations. Baseline categorical variables include: gender (male); gestational age (<32/40); side (right); tract (cingulum).

**Online Resource 11** Multiple regression results reporting the influence different variables on fractional anisotropy

| Variables included in the model | p-value |
| --- | --- |
| Gender | p=0.683 |
| Gestational age | **p=0.000** |
| PMA at scan | **p=0.000** |
| Side | p=0.829 |
| Tract:  CST  Fornix  ILF OR | **p=0.003**  p=0.155  p=0.737  p=0.272 |
| Gender x PMA | p=0.634 |
| Side x PMA | p=0.772 |
| PMA x GA | **p=0.000** |
| Tract x PMA:  CST  Fornix  ILF  OR | **p=0.006**  p=0.449  p=0.390  p=0.168 |
| Post-hoc tests for tracts x PMA:  Cingulum vs CST  Cingulum vs fornix  Cingulum vs ILF  Cingulum vs OR  CST vs fornix  CST vs ILF  CST vs OR  Fornix vs ILF  Fornix vs OR  ILF vs OR | **p=0.0057**  p=0.4488  p=0.3903  p=0.1685  **p=0.0001**  **p=0.0022**  **p=0.0000**  p=0.7665  p=0.4130  p=0.7622 |

* Significant, p<0.05, PMA: postmenstrual age at scan, CST: corticospinal tract, ILF: inferior longitudinal fasciculus, OR: optic radiations. Baseline categorical variables include: gender (male); gestational age (<32/40); side (right); tract (cingulum).
